# A predictive processing framework for body-oriented trauma intervention: a hypothesis illustrated by Body Connect Therapy

**DOI:** 10.3389/fpsyg.2026.1781289

**Published:** 2026-04-21

**Authors:** Masaki Fujimoto

**Affiliations:** School of Child Psychology, Tokyo Future University, Tokyo, Japan

**Keywords:** active inference, Body Connect Therapy, body-oriented psychotherapy, free energy principle, interoception, predictive processing, superior colliculus, trauma

## Abstract

This paper proposes a theoretical framework for understanding body-oriented trauma intervention by integrating predictive processing theory with candidate subcortical threat-processing pathways. Body-oriented interventions have gained increasing recognition in trauma treatment, yet the theoretical mechanisms underlying their effects remain underspecified. Within predictive processing, trauma is conceptualized as rigid, maladaptive predictions—particularly at the bodily level—that resist updating despite contradictory evidence. We identify the superior colliculus (SC) and its connections to the periaqueductal gray (PAG) and amygdala as candidate neural substrates for rapid, subcortical threat detection that may become dysregulated in trauma. The framework proposes four mechanisms through which body-oriented interventions may facilitate therapeutic change: (1) interoceptive attention increasing precision-weighting of bodily signals; (2) somatic safety experiences generating prediction errors that update threat models; (3) eye movement interventions potentially modulating SC-mediated defensive responses; and (4) “letting go” practices reducing excessive prior precision on threat predictions. Using Body Connect Therapy (BCT) as an illustrative case, we demonstrate how specific techniques may engage these theoretical mechanisms, while emphasizing that proposed mechanism-technique mappings remain hypothetical. The framework generates specific, testable predictions regarding interoceptive precision changes, SC pathway modulation, directional eye movement effects, and differential mechanism engagement across techniques. We discuss implications for understanding existing approaches, address alternative mechanisms for eye movement effects, and distinguish between common factors and technique-specific components. This theoretical integration aims to advance mechanistic understanding of body-oriented trauma intervention and guide future empirical research.

## 1 Introduction

Body-oriented interventions have gained increasing attention in the treatment of trauma and stress-related conditions, reflecting growing recognition of bodily processes in emotional regulation and psychological change (van der Kolk, 2014; Ogden et al., 2006). Approaches such as Somatic Experiencing (Levine, 1997), Sensorimotor Psychotherapy (Ogden et al., 2006), and other body-focused practices are now widely used clinically. However, despite expanding application, the theoretical mechanisms underlying these interventions remain underspecified. Clinical effectiveness has often preceded systematic theoretical clarification, leaving fundamental questions about how bodily processes contribute to psychological change largely unresolved.

Scope of “body-oriented interventions.” This paper focuses on interventions characterized by: (a) explicit direction of attention to bodily sensations during therapeutic work; (b) use of bodily experience (rather than verbal narrative alone) as a primary medium of processing; and (c) therapist-guided engagement with somatic responses to traumatic material. This includes approaches such as Somatic Experiencing, Sensorimotor Psychotherapy, and BCT, but excludes primarily behavioral interventions (e.g., exercise therapy), purely physiological interventions (e.g., vagus nerve stimulation), and contemplative practices (e.g., yoga, meditation) unless explicitly integrated into trauma-focused treatment. The boundaries are not sharp, and the framework may have varying applicability to related approaches.

Recent advances in predictive processing and related models of brain–body interaction offer a promising framework for addressing this gap (Clark, 2013; Friston, 2010; Seth, 2013; Linson and Friston, 2019; Putica and Agathos, 2024). Within this perspective, psychological distress arises from maladaptive predictive models and their associated precision-weighting processes, while therapeutic change involves updating these models through embodied experience. The superior colliculus-periaqueductal gray (SC-PAG) pathway, a phylogenetically ancient threat detection and defensive response system, provides one candidate neuroanatomical substrate for understanding how bodily interventions may modulate subcortical threat processing (Dean et al., 1989; Evans et al., 2018).

This paper articulates a conceptual framework for understanding body-oriented interventions through integrating predictive processing theory with SC-mediated pathways, focusing on how bodily engagement may facilitate changes in perception, affect regulation, and subjective experience. To concretize this proposal, Body Connect Therapy (BCT) serves as an illustrative case. BCT is a body-oriented approach developed in Japan that works explicitly with bodily sensation, attention, and regulation in trauma-related contexts (Fujimoto, 2019). This paper does not aim to establish BCT’s superiority or general efficacy, nor to propose a comprehensive theory for all body-oriented interventions. Rather, BCT exemplifies how specific techniques may map onto broader theoretical principles.

## 2 Theoretical framework

### 2.1 Predictive processing: the brain as a prediction machine

Contemporary neuroscience has reconceptualized the brain not as a passive information processor but as an active prediction machine (Clark, 2013; Friston, 2010). The brain continuously generates predictions about incoming sensory information and compares them with actual input. The discrepancy—termed prediction error—serves as a learning signal driving internal model updating.

Rao and Ballard (1999) provided an influential computational model of hierarchical predictive coding in visual cortex, demonstrating how top-down predictions and bottom-up prediction errors exchange across cortical layers. This framework has since been extended to encompass perception, action, learning, and attention as manifestations of a single principle: prediction error minimization, or equivalently, free energy minimization (Friston, 2010).

The brain reduces prediction error through two complementary mechanisms. Perceptual inference updates internal models to match sensory input—changing beliefs about the world. Active inference acts upon the environment to make sensory input conform to predictions—changing the world to match beliefs (Friston, 2010). Both serve the imperative of maintaining accurate predictive models. Recent advances have demonstrated the utility of predictive coding and active inference models for understanding psychopathology and informing clinical interventions (Smith et al., 2021).

### 2.2 Precision-weighting and attention

Not all prediction errors are treated equally. Precision-weighting refers to the brain assigning different confidence levels to different information sources (Feldman and Friston, 2010). Prediction errors from precise (reliable) sources are weighted heavily, influencing model updating more strongly, while imprecise signals are down-weighted. Terminological clarification. In predictive processing, “precision” can refer to several distinct quantities: (a) likelihood precision—the estimated reliability of sensory prediction errors; (b) prior precision—confidence in prior beliefs; and (c) in active inference frameworks, policy precision—confidence in action selection. Throughout this paper, we use precision in the following specific senses: when discussing interoceptive attention (Mechanisms 1 and 4), we refer primarily to likelihood precision of bodily signals; when discussing rigid threat predictions in trauma (Section 2.4), we refer to prior precision of threat-related beliefs. We acknowledge that these distinctions are sometimes conflated in the clinical literature, and precise computational specification remains an important direction for future theoretical development.

Table 1 summarizes these precision distinctions and their clinical/therapeutic implications.

**Table 1:** Precision types and their roles in the proposed framework.

Clinical presentation	Precision type	Proposed dysregulation
Hyperarousal	Likelihood precision (interoceptive)	Excessive: threat-related bodily signals over-weighted
Dissociation	Likelihood precision (interoceptive)	Reduced: bodily signals under-weighted (contested; see Section 2.4)
Rigid threat beliefs	Prior precision	Excessive: threat predictions resist updating
Mechanism 1 (Interoceptive attention)	Likelihood precision	Therapeutic increase through attentional focus
Mechanism 4 (Top-down relaxation)	Prior precision	Therapeutic decrease enabling model updating

This summary represents the primary precision type implicated in each case; actual clinical presentations involve complex interactions across precision types.

Attention can be understood as precision-weighting optimization (Hohwy, 2012). Attending to a stimulus or bodily region increases precision-weighting of signals from that source (likelihood precision), making them more influential in prediction error computation. This has important implications for body-oriented interventions: directing attention to bodily sensations may fundamentally alter interoceptive signal precision-weighting.

### 2.3 Interoception and bodily predictions

Predictive processing applies not only to exteroception but also to interoception—sensing internal bodily states (Seth, 2013; Barrett and Simmons, 2015). The brain continuously predicts interoceptive signals such as heartbeat, respiration, muscle tension, and visceral sensations, comparing predictions with actual afferent input.

Seth (2013) proposed interoceptive inference, suggesting emotional experience arises from the brain’s predictions about interoceptive signal causes. Feelings are not simply bodily state readouts but are actively constructed through predictive modeling—with profound implications for understanding emotional disorders and their treatment.

Crucially, interoceptive signal precision-weighting appears malleable. Practices directing attention to bodily sensations—including body-oriented psychotherapies—may increase interoceptive prediction precision-weighting, thereby enhancing bodily signal influence on overall predictive processing (Paulus and Stein, 2010).

### 2.4 Trauma as rigid, maladaptive predictions

Within predictive processing, trauma can be conceptualized as involving rigid, high-precision beliefs that resist updating despite contradictory evidence (Kube et al., 2020; Putica and Agathos, 2024; Linson and Friston, 2019). Within the active inference framework specifically, Linson et al. (2020) model trauma as excessively precise prior aversions—strong expectations about outcomes that must be avoided—which generate chronic defensive responding even in safe environments by lowering the threshold for exploitation over exploration—that is, shifting the system toward using established threat predictions rather than exploring new, potentially safety-relevant information. Following traumatic experiences, the brain may develop strong prior beliefs—particularly regarding threat and safety—assigned very high precision, thus dominating perception and behavior.

For example, someone who experienced interpersonal violence may develop a high-precision prediction that “other people are dangerous.” This prediction, encoded at multiple hierarchy levels including the bodily level, generates persistent defensive mobilization (Porges, 2011). The high precision assigned to this threat prediction means even repeated safety experiences may fail to generate sufficient prediction error for model updating (Kube et al., 2020).

At the bodily level, traumatic experiences establish maladaptive interoceptive predictions (Paulus and Stein, 2010; Khalsa et al., 2018). The body “expects” danger and generates corresponding defensive states (elevated heart rate, muscle tension, hypervigilance) even in objectively safe situations. These bodily predictions may particularly resist verbal or cognitive intervention because they operate at hierarchy levels not directly accessible to linguistic processing (van der Kolk, 2014).

Predictive processing accounts of trauma suggest that extreme events generate unusually large prediction errors that cannot be readily resolved within the existing hierarchical generative model of the self. When the discrepancy between prior expectations and incoming sensory signals becomes overwhelming, the system may fail to integrate the experience into coherent autobiographical memory.

Recent work has proposed that dissociation may reflect a functional decoupling between lower-level sensory representations and higher-level self-models within the predictive hierarchy (Wilkinson et al., 2017), leaving traumatic experiences encoded as fragmented sensory-affective traces. To manage persistent uncertainty, the brain may recalibrate toward rigid, hyperprecise threat predictions (Krupnik, 2020).

These somatically fixed, fragmented representations constitute a primary target of body-oriented interventions such as BCT, which aim to generate corrective prediction errors at the bodily level rather than through top-down verbal processing.

Importantly, precision dysregulation in trauma may manifest bidirectionally: hyperarousal states involve excessive likelihood precision of threat-related interoceptive signals, while dissociative states may involve pathologically reduced precision-weighting of bodily signals (cf. Seth and Friston, 2016; Paulus et al., 2019). However, we note that the computational account of dissociation remains contested. Alternative or complementary interpretations include: (a) attenuated prior precision for bodily self-models rather than likelihood precision; (b) disrupted attentional allocation (policy precision) preventing interoceptive engagement; (c) metacognitive deficits in awareness of interoceptive states; or (d) active suppression as a regulatory strategy rather than passive signal attenuation. These alternatives have different implications for intervention and require empirical differentiation. These differential presentations may generate distinct predictions for intervention: in hyperarousal-predominant PTSD, gradual titration of interoceptive attention may be prioritized to avoid overwhelming already elevated precision; in the dissociative subtype, initial work may focus on re-establishing basic interoceptive connectivity before processing is attempted. Empirical validation of these differential predictions represents an important direction for future research.

This bidirectional model, whatever its precise computational implementation, has implications for intervention: hyperarousal may require interventions that reduce the gain on threat signals, while dissociation may require interventions that increase interoceptive engagement to re-establish bodily awareness. Body-oriented interventions may need to address these presentations differently—a clinical intuition consistent with trauma therapy practice (Ogden et al., 2006) but requiring systematic empirical investigation.

This account also bears on the question of how cognitive therapies work for trauma—a seemingly paradoxical result if bodily predictions resist verbal intervention. We suggest that effective cognitive therapies may work partly by altering the precision-weighting of linguistic versus interoceptive channels, or by generating prediction errors through behavioral experiments that engage bodily systems. The relationship between cognitive and body-oriented mechanisms deserves systematic investigation.

### 2.5 Superior colliculus pathways: potential neural substrates for threat processing

While predictive processing provides a computational account of trauma and recovery, understanding neural substrates requires attention to specific circuits. The superior colliculus (SC) serves as a hub connecting to multiple downstream systems relevant to threat processing. We focus on SC rather than other subcortical routes (e.g., thalamo-amygdala pathway) because the SC uniquely integrates threat detection with eye movement control—providing a direct anatomical link to eye movement-based interventions that other pathways lack. Notably, the thalamo-amygdala pathway has been implicated in EMDR theorizing (Bergmann, 2008), but this route does not directly explain why directional eye movements might produce differential effects—a gap that the SC’s role in spatial attention and orienting may address.

The superior colliculus, located in the midbrain, integrates visual, auditory, and somatosensory information to detect environmental threats (Stein and Meredith, 1993; May, 2006). Operating largely outside conscious awareness, it triggers defensive responses within milliseconds of threat detection (Evans et al., 2018). This millisecond-scale processing may help explain why verbal and cognitive interventions which operate on slower timescales requiring cortical elaboration may be insufficient to modify threat responses that are initiated before conscious awareness. Two downstream pathways are particularly relevant to trauma processing. First, the SC projects directly to the periaqueductal gray (PAG), which orchestrates autonomic and behavioral defensive response components including freezing, flight, and fight (Bandler and Shipley, 1994; Fanselow, 1994; Dean et al., 1989). Second, one recent high-field fMRI study reported functional connectivity evidence consistent with an SC-pulvinar-amygdala pathway in humans, with activity patterns correlating with the intensity of negative emotional responses (Kragel et al., 2021). This rapid subcortical route may complement cortical processing of affective information, though its precise functional role continues to be debated and replication in independent samples is needed.

Importantly, the SC is intimately involved in eye movement control. Saccadic eye movement circuits overlap substantially with those for threat detection and orienting (Krauzlis et al., 2013; Gandhi and Katnani, 2011). This anatomical fact suggests a potential mechanism for eye movement interventions: voluntary eye movements may engage the SC in ways that modulate both defensive (SC-PAG) and affective (SC-pulvinar-amygdala) processing. While SC-mediated pathways are emphasized here, voluntary eye movements are also strongly regulated by the Frontal Eye Fields (FEF), which maintain connections with multiple brainstem regions. FEF-mediated top-down control may therefore also contribute to the therapeutic effects of intentional eye movements in BCT, alongside SC-mediated bottom-up modulation. Full characterization of the relative contributions of SC and FEF pathways remains an important direction for future research.

In trauma, these SC-mediated pathways may become hyperactivated, maintaining chronic threat detection even in safe environments (Lanius et al., 2017). Functionally, these subcortical systems can be construed as rapid defensive circuits that, when hyperactivated, generate persistent threat-related responses contributing to hypervigilance, exaggerated startle, and autonomic dysregulation in trauma-related disorders (Lanius et al., 2017; Porges, 2011). We note that Polyvagal Theory, while influential in clinical trauma literature, has been critiqued on phylogenetic and physiological grounds (Grossman and Taylor, 2007); our framework does not depend on Polyvagal-specific claims but draws more broadly on established defensive circuit neuroscience.

It should be noted that bodily predictions are not exclusively generated at the level of the SC. Rather, they are distributed across multiple hierarchical levels, including the brainstem, cerebellum, insular cortex, and higher cortical regions. The SC is particularly associated with rapid orienting and threat detection, while the insula serves as a primary site for interoceptive prediction and error signaling. The present framework emphasizes SC-PAG pathways as a primary entry point for body-oriented intervention, while acknowledging that these pathways operate within a broader hierarchical network of bodily prediction generation.

The cerebellum likely participates in the predictive updating of bodily states by contributing to sensorimotor and interoceptive error correction (Ito, 2008; Sokolov et al., 2017). Disruption to cerebello-thalamo-cortical circuits during traumatic memory retrieval, as demonstrated by Kearney et al. (2025), suggests that impaired cerebellar predictive updating may contribute to the fragmented and sensorially intense quality of traumatic memory in PTSD. How BCT and related body-oriented interventions engage cerebellar predictive mechanisms remains an important direction for future research.

### 2.6 Integrating predictive processing with SC-mediated subcortical pathways: a candidate account

A critical question arises regarding the relationship between predictive processing—a computational-level account—and SC-mediated pathways—an implementation-level description. We must be explicit about the nature, limits, and epistemic status of this integration.

Epistemic status. The SC-pathway account proposed here should be understood as one candidate mechanism among several (see Section 4.3 for alternatives), not as an established explanation. Its value lies primarily in generating distinguishable predictions that can advance understanding of eye movement effects in trauma therapy—predictions that differ from those of working memory or interhemispheric accounts. We do not claim that SC modulation is the primary or sole mechanism; rather, we propose it as a testable hypothesis worthy of empirical investigation.

Predictive processing, particularly in its hierarchical predictive coding formulation, has been primarily developed for cortical systems (Rao and Ballard, 1999; Friston, 2010). However, the broader framework of active inference (Friston et al., 2017; Parr and Friston, 2017) extends naturally to reflexive and subcortical systems. Active inference proposes that all biological systems minimize free energy through action and perception, without requiring explicit hierarchical prediction computation. Within this formulation, SC-mediated defensive responses can be understood as embodying implicit “expectations” about environmental threats—not through cortical-style prediction error computation, but through evolutionarily tuned sensorimotor contingencies that influence precision-weighting at higher levels.

We emphasize that this integration represents a functional re-description with acknowledged limitations: we are not claiming that the SC literally “computes predictions” in the manner of cortical hierarchies. Rather, we propose that SC-related circuitry can be construed as supporting rapid orienting and defensive control loops that (a) operate with high gain (functionally analogous to high likelihood precision), (b) generate afferent signals that cortical predictive systems must accommodate, and (c) are themselves subject to modulation by descending cortical predictions (cf. Kanai et al., 2015). This bidirectional interaction—rather than subcortical “predictions” per se—may be the key mechanistic interface, though this remains speculative.

Within this candidate framework, traumatic experiences may establish defensive response patterns at the subcortical level that resist updating through higher-level cognitive processes. The SC-PAG system may continue generating threat-related afferent signals even when cortical systems have access to safety information. This creates a situation where cortical prediction error minimization mechanisms must continuously accommodate persistent “threat signals” from below—potentially maintaining high-precision threat priors (prior precision) through bottom-up constraint (i.e., threat predictions generated at subcortical levels that are resistant to revision by cortical safety signals). This mismatch between subcortical defensive activation and cortical safety evaluations may contribute to the phenomenology of trauma: individuals “know” they are safe but “feel” endangered (van der Kolk, 2014; Porges, 2011).

This raises an important conceptual question: how do cortical systems obtain safety information if sensory signals are first processed through subcortical pathways? Two complementary mechanisms may be at work. First, cortical systems do receive safety-relevant sensory information, but defensive circuits operating at the subcortical level may bias its interpretation, prioritizing threat-consistent evidence and suppressing safety-relevant signals (mechanism a). Second, cortical systems may infer safety based on contextual knowledge and declarative memory rather than direct sensory evidence, while subcortical circuits continue to generate defensive responses based on deeply encoded threat predictions (mechanism b). This distinction helps clarify the clinically familiar phenomenon of “knowing you are safe but feeling unsafe.”

Human functional connectivity evidence suggests the existence of an SC-pulvinar-amygdala pathway that may contribute to rapid affective salience encoding (Kragel et al., 2021). This pathway may influence precision-weighting at early processing stages before cortical elaboration, though its precise functional role and relevance to clinical intervention remain open questions.

If the SC-pathway account has merit, then interventions directly engaging these systems—such as those involving eye movement—may be relevant because they target the source of bottom-up constraint on cortical predictions. However, this remains a hypothesis requiring empirical validation, and alternative mechanisms (Section 4.3) may prove equally or more important.

### 2.7 Mechanisms of body-oriented intervention: a theoretical proposal

**Figure 1:**
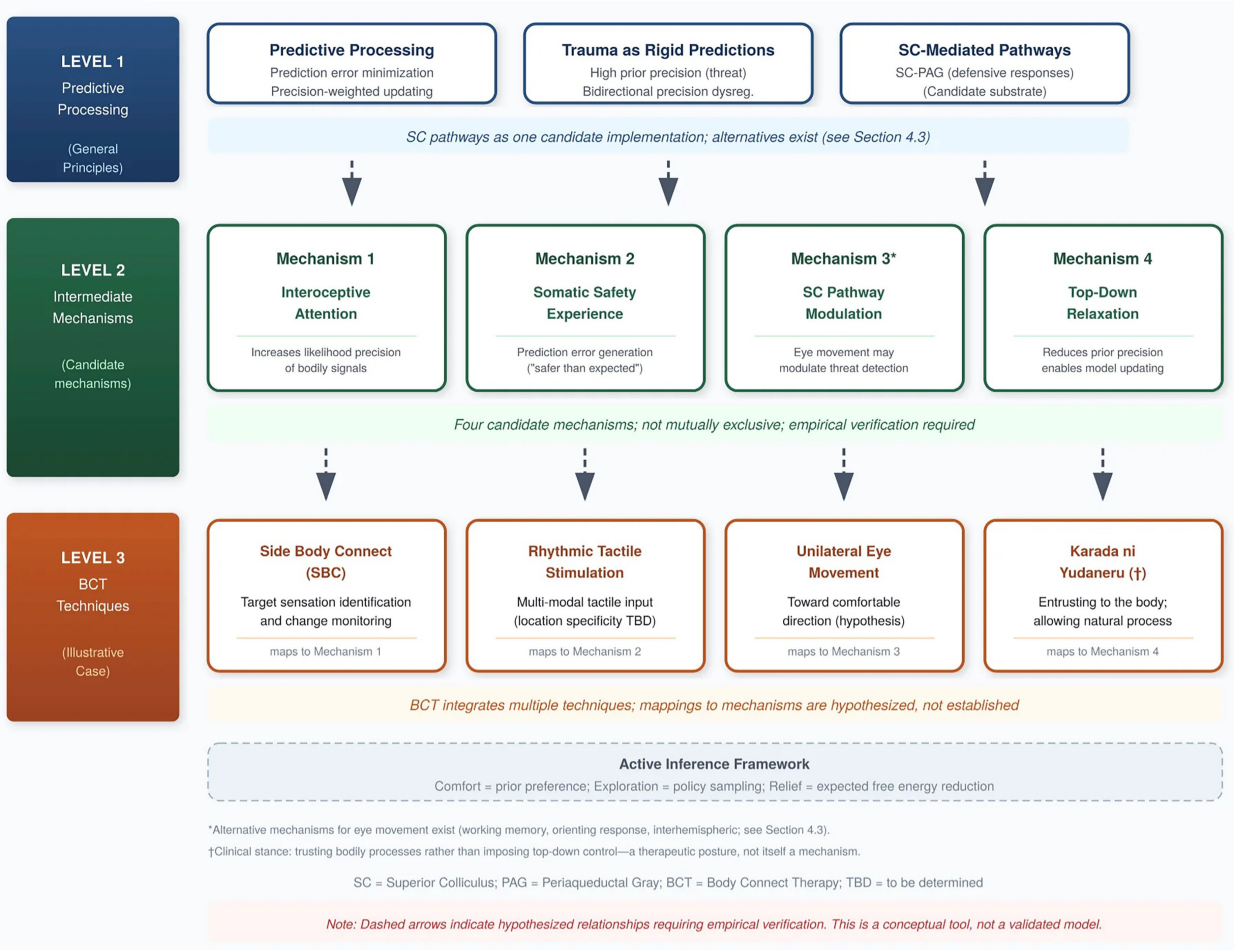
Predictive processing framework for body-oriented trauma intervention. Level 1: General principles (predictive processing, trauma as rigid predictions, SC-mediated pathways). Level 2: Four candidate mechanisms (interoceptive attention, somatic safety experience, SC pathway modulation, top-down relaxation). Level 3: BCT techniques as illustrative implementation. Dashed arrows indicate hypothesized relationships requiring empirical verification. SC = Superior Colliculus; PAG = Periaqueductal Gray.

Based on this analysis, we propose that body-oriented trauma interventions operate through four interrelated mechanisms (see Figure 1):

#### 2.7.1 Mechanism 1: Interoceptive attention and precision reweighting

Body-oriented interventions characteristically direct attention to bodily sensations (Ogden et al., 2006; Levine, 1997). This attentional focus increases interoceptive signal precision-weighting, making them more influential in prediction error computation (Ainley et al., 2016; Farb et al., 2015). This may facilitate bodily prediction updating by amplifying actual bodily state signals relative to top-down predictions. It is important to acknowledge that directing attention to bodily sensations is not universally applicable; in cases of severe PTSD, interoceptive signals may be overwhelming rather than underweighted, risking amplification of distress rather than generating corrective prediction errors. BCT addresses this challenge through the SCRIPT model, in which Titration—approaching traumatic material in small, manageable increments within the client’s window of tolerance—is central (Fujimoto, 2026). Resource-building and somatic safety establishment precede trauma processing, preventing re-traumatization.

#### 2.7.2 Mechanism 2: Somatic safety experience and prediction error generation

Whereas Mechanism 1 concerns the modulation of interoceptive precision through attentional deployment, Mechanism 2 describes the generation of novel prediction errors through the introduction of safety-relevant bodily experiences—these are conceptually distinct processes that may operate sequentially or in parallel during BCT. Many body-oriented interventions facilitate bodily safety, relaxation, or comfort experiences during trauma-related activation (Levine, 1997; Ogden et al., 2006; Porges, 2011). When clients “expecting” continued distress during trauma recall instead experience unexpected relief or calm, significant prediction error is generated. This prediction error—“I expected to feel terrible, but I feel okay”—provides the learning signal for updating maladaptive predictions (Ecker et al., 2024; Lane et al., 2015). From a predictive processing perspective, “safety” can be defined computationally as a state in which prediction errors are minimal and the posterior distribution over bodily states has appropriately calibrated precision—neither hypervigilant nor dissociated, but flexibly responsive to actual environmental contingencies. The assumption that comfort serves as a prior preference for safety-related bodily states may not hold for individuals with severe developmental trauma, whose prior expectations may be organized around chronic threat or dysregulation rather than safety—that is, individuals who may have limited experiential knowledge of what physiological comfort or safety feels like. In such cases, BCT begins not with activating pre-existing comfort priors, but with gradually constructing new safety-relevant prior distributions through extended somatic resource-building—a theoretically distinct mechanism warranting further empirical investigation.

#### 2.7.3 Mechanism 3: SC pathway modulation through eye movement

Eye movement interventions may engage the superior colliculus, potentially modulating both downstream pathways relevant to trauma processing. Given the SC’s established role in saccade generation and orienting/attention control (Gandhi and Katnani, 2011; Krauzlis et al., 2013), voluntary eye movements—particularly toward comfortable or preferred directions—may modulate SC activity and reduce defensive output. This possibility has been discussed in the context of EMDR and related approaches (Bergmann, 2008; Gunter and Bodner, 2008). One possible interpretation is that this modulation may affect two parallel systems: (1) SC-PAG circuitry, potentially attenuating defensive mobilization indexed by startle reflex, heart rate variability, and skin conductance; and (2) SC-pulvinar-amygdala circuitry, potentially altering the processing of incoming threat-related sensory signals before they reach cortical awareness (cf. Kragel et al., 2021; Lanius et al., 2017). Both pathways may contribute to creating conditions conducive to therapeutic change, though this remains speculative.

#### 2.7.4 Mechanism 4: Relaxation of top-down predictive control

Body-oriented interventions emphasizing “letting go” or “allowing” bodily processes to unfold naturally may facilitate relaxing rigid top-down predictions (Gendlin, 1996; Levine, 1997). By reducing precision assigned to prior beliefs and increasing openness to bottom-up sensory information, these interventions may create conditions enabling maladaptive prediction updating by current experience (Farb et al., 2015; Hölzel et al., 2011). These four mechanisms constitute a theoretical framework for understanding body-oriented intervention in trauma processing. They are not mutually exclusive; effective interventions likely engage multiple mechanisms simultaneously. The framework serves as a conceptual tool for organizing clinical observations and guiding future research, not as an empirically established model.

## 3 BCT as an illustrative CASE

### 3.1 Overview of Body Connect Therapy

Body Connect Therapy (BCT) is a body-oriented trauma treatment approach developed in Japan that integrates eye movement with acupoint stimulation (Fujimoto, 2019). Central to BCT is the organizing concept karada ni yudaneru (身体に委ねる), translating as “entrusting to the body.” This phrase captures BCT’s fundamental clinical stance: trusting bodily sensations, reactions, and directions, and returning agency to the body itself.

BCT rests on three core principles: (1) the body leads—therapeutic change emerges from bodily processes rather than cognitive analysis; (2) the body feels safety— intervention aims to establish somatic safety experiences; and (3) natural healing capacity operates—the therapist facilitates rather than directs the body’s inherent self-regulation capacity.

The primary BCT technique is Side Body Connect (SBC). In SBC, clients first recall traumatic material and identify a target body sensation—often tension, discomfort, or activation. To determine the “comfortable direction,” clients close their eyes and slowly move them horizontally left and right, noticing which direction feels more comfortable. In this paper, “comfortable direction” refers to the gaze direction subjectively reported as easier or more pleasant during slow horizontal exploration, assessed moment-to-moment rather than as a stable trait. Once this preferred direction is established, clients open their eyes and perform slow, unilateral eye movements tracking a pointer (or therapist’s fingers) toward that comfortable direction. During eye movement, the client simultaneously applies gentle tapping stimulation to specific acupoints on their own hand or body (commonly including points such as Hegu/LI4), as directed by the therapist. Clients are encouraged to simply allow and experience whatever naturally arises—embodying “entrusting to the body.”

### 3.2 Mapping BCT techniques onto theoretical mechanisms

For readers unfamiliar with Body Connect Therapy (BCT), the Side Body Connect (SBC) technique typically involves inviting clients to attend to bodily sensations associated with distress, followed by gentle eye movements and tactile stimulation oriented toward a subjectively comfortable direction. This paper does not aim to specify procedural details, but to clarify the theoretical principles such interventions may instantiate.

BCT techniques map onto the four proposed mechanisms (see Figure 1):

Side Body Connect (SBC) → Interoceptive Attention

In SBC, clients attend to bodily sensations including tension, discomfort, or activation related to traumatic material. This focused interoceptive attention may increase bodily signal precision-weighting (Farb et al., 2015; Mehling et al., 2012), amplifying bottom-up input relative to top-down predictions and facilitating maladaptive bodily prediction updating (Seth, 2013; Critchley and Garfinkel, 2017).

Unilateral Eye Movement Toward Comfort → SC-PAG Pathway Modulation + Top-Down Relaxation

BCT’s distinctive slow, unilateral eye movement toward subjectively comfortable directions may engage the SC-PAG system differently from bilateral eye movements in other approaches (cf. Shapiro, 2018). Movement toward comfort, rather than alternating bilateral movement, may be experienced as safety-consistent and could potentially attenuate hyperactivated SC-PAG circuitry (Lanius et al., 2017). Simultaneously, moving toward what feels comfortable embodies “entrusting to the body,” potentially reducing rigid top-down predictive control (cf. Levine, 1997).

Theoretical basis for directional preferences. Why might traumatic experiences generate directional gaze preferences at all? We acknowledge that this construct is currently under-theorized, but several considerations suggest plausibility. First, the SC integrates multisensory information with motor maps for orienting (Stein and Meredith, 1993), and threat-related experiences may establish asymmetric activation patterns in these maps. Second, individual differences in hemispheric lateralization for emotional processing (Gainotti, 2012) might contribute to directional preferences, though this would predict stable rather than state-dependent preferences. We do not claim that comfortable direction specifically reflects “safety processing” as opposed to these other factors; rather, we propose this as a testable hypothesis.

It should be noted that the subjective sense of “comfort” associated with a particular gaze direction could reflect multiple factors beyond threat-safety processing. Alternative explanations include: (1) oculomotor ease or reduced muscle strain in certain directions; (2) pre-existing attentional biases or hemispheric asymmetries; (3) vestibular contributions, which—rather than representing a mutually exclusive alternative—may be integral to safety processing given the vestibular system’s tight connections with both SC and cerebellar circuits involved in spatial orientation and autonomic regulation; or (4) simple habituation or familiarity effects. The hypothesis that comfortable-direction preference specifically reflects safety-related processing, rather than these alternatives, can be empirically distinguished through the paradigm described in Prediction 5 below.

Stability versus state-dependence. Clinical observation suggests that comfortable direction may shift during therapeutic processing, with some clients reporting that the initially preferred direction becomes less comfortable as processing continues. This state-dependence—if confirmed empirically—would argue against purely oculomotor or stable hemispheric explanations and would be more consistent with the hypothesis that directional preference reflects current threat-safety processing state. However, we lack systematic data on when and why such shifts occur, representing an important gap for future research.

From a predictive processing perspective, the rationale for unilateral comfortable-direction movement differs fundamentally from bilateral alternation. Whereas bilateral stimulation may function primarily through working memory taxation or interhemispheric communication (Gunter and Bodner, 2008; van den Hout and Engelhard, 2012), unilateral movement toward comfort can be interpreted as one possible form of active inference.

Active inference formulation. To make this connection explicit: in active inference terms, (a) “comfort” corresponds to a prior preference (the agent’s expectation of preferred bodily states—low arousal, safety); (b) the exploratory gaze movement constitutes policy sampling (testing which action leads to preferred outcomes); and (c) the resulting sense of calm or relief reflects expected free energy reduction—a decrease in both uncertainty about the current state and distance from preferred states. The client’s selection of comfortable direction can thus be understood as implicit policy optimization: choosing the action (gaze direction) that minimizes expected free energy. This formulation remains speculative and requires empirical testing, but it provides a computational vocabulary for the clinical observation that “following the body’s preference” appears therapeutically relevant. This formulation differs from simple reinforcement learning accounts in a crucial respect: rather than merely maximizing reward or minimizing aversive outcomes through trial-and-error, active inference agents minimize expected free energy by considering both epistemic value (reducing uncertainty about the world) and pragmatic value (achieving preferred states). The comfortable-direction selection may thus involve not only seeking safety but also resolving uncertainty about one’s own bodily state—a distinctively active inference prediction. We acknowledge, however, that at the behavioral level, distinguishing between active inference and reinforcement learning accounts may prove empirically challenging; both frameworks can often accommodate the same behavioral observations through different parameterizations. The subjective experience of “relief” during BCT can be computationally interpreted as expected free energy reduction—the agent’s prediction that continuing the current action policy (gaze toward comfort) will minimize both sensory prediction error and the divergence from preferred bodily states. This accounts for why relief often precedes measurable physiological change: the reduction in expected free energy is computed prospectively.

A parsimonious interpretation is that sustained gaze toward a self-selected “safe” direction may bias the system toward safety-consistent inference, effectively reweighting the predictive hierarchy away from threat expectations. This hypothesis— that direction-specific eye movement selectively modulates threat-versus-safety predictions—remains empirically untested but generates specific predictions (see Section 4.5).

Rhythmic Tactile Stimulation → Somatic Safety Experience

During BCT, the client applies rhythmic tapping stimulation to their own hand or body as directed by the therapist. In clinical practice, this is typically applied to traditional acupoints (e.g., Hegu/LI4), though the theoretical rationale for acupoint specificity versus general tactile stimulation remains unclear. We therefore describe this component as “rhythmic tactile stimulation” and treat acupoint specificity as an open empirical question rather than a theoretical claim.

Rhythmic tactile stimulation may contribute to somatic safety experiences through multiple pathways within the predictive processing framework: (1) as a source of predictable, rhythmic somatosensory input that may be experienced as safe and non-threatening, thereby generating prediction errors when expected distress fails to materialize; (2) as an additional modality that increases the salience of bodily signals, potentially enhancing interoceptive likelihood precision; or (3) through direct autonomic effects (e.g., vagal activation via mechanoreceptor stimulation) that alter the interoceptive signals available for cortical prediction. Research on acupoint stimulation suggests limbic and autonomic modulation (Fang et al., 2009; Hui et al., 2010), though the specificity of these effects to acupoint versus non-acupoint locations has not been definitively established. Whether acupoint-specific stimulation produces effects beyond those of general rhythmic tactile stimulation remains an empirical question requiring dismantling studies (see Prediction 7). Until such evidence is available, we make no claim for acupoint specificity and treat this as an area of uncertainty in the current framework.

Multi-modal stimulation—combining visual (eye movement), interoceptive (body awareness), and somatosensory (tactile) channels—may amplify prediction errors when expected distress fails to materialize (cf. Stein and Meredith, 1993). However, we acknowledge uncertainty about whether these inputs would sum synergistically, act independently, or potentially interfere. The conditions under which multi-modal input enhances versus complicates therapeutic processing remain to be determined empirically.

### 3.3 Clinical observations: consistency, limitations, and potential disconfirmation

Clinical illustrations of the mechanisms proposed in this framework are provided in a companion case report (Fujimoto, 2026), documenting BCT applications across two contrasting presentations: an acute stress reaction resolved in four sessions, and a complex developmental trauma case treated over 3 years. These cases demonstrate titration strategies for clients with overwhelming interoceptive signals and BCT’s approach to developmental trauma where safety priors are absent.

Clinical observations in BCT practice can be organized in relation to the theoretical framework, while acknowledging significant methodological limitations.

Observations consistent with the framework. Clients frequently report experiences such as “I’m thinking about the memory, but my body feels calmer than I expected” or “The tightness in my chest is releasing even though I’m still remembering.” These reports are consistent with prediction error generation—discrepancies between expected and experienced bodily states. The relatively rapid change sometimes observed in BCT sessions could reflect simultaneous engagement of multiple mechanisms.

In some cases, as processing continues, clients report that the initially comfortable gaze direction becomes less preferred, with the neutral (central) position feeling more comfortable. This shift, when it occurs, could reflect dynamic changes in threat-safety processing, though alternative explanations (fatigue, habituation) cannot be ruled out.

Critical methodological limitations. These observations must be interpreted with extreme caution and should not be treated as evidence for the framework’s validity. The limitations are severe: (1) Post-hoc consistency is weak evidence-virtually any therapeutic change can be re-described in predictive processing terms, and the observations were not designed to test the framework. (2) The observations come from the BCT developer treating clients with BCT, creating substantial risk of expectation effects and interpretation bias; independent replication by researchers without conflicts of interest is essential. (3) No systematic comparison with alternative frameworks has been conducted. (4) Sample characteristics, treatment parameters, and outcome measures are not standardized.

What would disconfirming observations look like? The framework would be challenged by observations such as: (a) clients consistently reporting increased bodily distress during comfortable-direction eye movement (contrary to the safety-processing hypothesis); (b) no relationship between reported “surprise” at feeling better than expected and therapeutic progress; (c) comfortable-direction preference showing no state-dependence across sessions (suggesting stable trait rather than threat-related processing); or (d) clients with high interoceptive accuracy showing worse rather than better outcomes (contrary to the precision-reweighting account).

We emphasize that these clinical observations do not constitute empirical validation. The theoretical account serves as a conceptual tool for organizing observations and generating testable hypotheses, not as an established explanation of BCT’s mechanism of action. Systematic research with appropriate controls and blinding is essential.

## 4 Discussion

### 4.1 Theoretical contributions

This paper proposed a theoretical framework for understanding body-oriented trauma interventions by integrating predictive processing theory with SC-mediated subcortical pathways (SC-PAG and SC-pulvinar-amygdala). Four mechanisms—interoceptive attention, somatic safety experience, SC pathway modulation, and top-down control relaxation—are proposed as intermediate principles potentially common to various body-oriented approaches.

The framework offers several contributions. First, it situates body-oriented approaches within contemporary computational neuroscience (Clark, 2013; Friston, 2010), providing principled account of why bodily engagement may be necessary for trauma processing (cf. Price and Hooven, 2018). Second, it integrates cognitive/computational (predictive processing) and neuroanatomical (SC-mediated subcortical circuits) analysis levels (cf. Marr, 1982), offering a more complete mechanistic picture. Third, it generates specific, testable hypotheses about processes underlying therapeutic change (cf. Kazdin, 2007).

### 4.2 Implications for other body-oriented approaches

Although BCT served as the illustrative case, the proposed framework may have broader applicability. Other body-oriented approaches—Somatic Experiencing (Levine, 1997; Payne et al., 2015), Sensorimotor Psychotherapy (Ogden et al., 2006), and related methods (Price and Hooven, 2018)—share features including attention to bodily sensation, somatic safety facilitation, and emphasis on allowing bodily processes to unfold. The four mechanisms may provide common theoretical language for understanding diverse body-oriented interventions (cf. Röhricht, 2009).

This does not imply all body-oriented approaches operate identically; different techniques may emphasize different mechanisms. The framework may serve as a tool for analyzing specific mechanisms engaged by different interventions, potentially informing more targeted and effective treatment development (cf. Holmes et al., 2018).

The present framework also shares theoretical ground with Deep Brain Reorienting (DBR; Corrigan and Christie-Sands, 2020), a trauma therapy model explicitly grounded in SC-mediated orienting responses. DBR’s therapeutic sequence begins with orienting tension—fleeting muscular tension around the eyes, forehead, or back of the neck—hypothesized to reflect SC-initiated preparatory motor responses to salient stimuli. A randomized controlled trial demonstrated significant reductions in PTSD symptom severity following eight DBR sessions (Cohen’s *d* = 1.17 at post-treatment), with notably low dropout rates (Kearney et al., 2023), providing preliminary empirical support for SC-targeted therapeutic approaches. BCT similarly engages SC-mediated pathways through slow, unilateral eye movements, while integrating acupoint stimulation and body-focused attention. Both approaches converge on the principle that engagement with subcortical orienting circuits prior to cortical processing may be important in resolving trauma-related threat predictions that are resistant to top-down verbal intervention.

### 4.3 Alternative mechanisms for eye movement effects: comparison with the present account

The therapeutic effects of eye movement in trauma treatment have generated multiple competing explanations, and intellectual honesty requires explicit engagement with these alternatives. The present SC-modulation account must be evaluated against established alternative mechanisms.

Working memory taxation hypothesis. Van den Hout and Engelhard (2012) propose that eye movements during trauma recall create dual-task demands that compete for limited working memory resources, reducing the vividness and emotionality of traumatic memories. This account is supported by substantial experimental evidence and does not require SC involvement. However, working memory taxation predicts that any cognitively demanding task should produce similar effects—a prediction only partially supported by comparative studies. Moreover, this account does not straightforwardly explain why slow, unilateral eye movements (as in BCT) would be effective, since the dual-task demands would presumably be lower than rapid bilateral movements. Notably, the working memory account predicts dose–response relationships with cognitive load—more demanding tasks should produce stronger effects. The SC-modulation account does not necessarily make this prediction; instead, it predicts that direction-specific effects should be observed regardless of cognitive demand level, as the mechanism operates through subcortical rather than working memory pathways. Orienting response and dearousal. Armstrong and Vaughan (1996) suggested that eye movements trigger orienting responses that compete with and attenuate fear states. This account shares some features with the present framework (both emphasize subcortical involvement in orienting) but differs in proposed mechanism—dearousal through response competition versus modulation of threat-processing circuitry.

Interhemispheric interaction hypothesis. Propper and Christman (2008) proposed that bilateral stimulation enhances interhemispheric communication, facilitating episodic memory processing. This account specifically predicts advantages for bilateral over unilateral stimulation—the opposite of what BCT proposes. If BCT’s unilateral movement proves effective, this would constitute evidence against the interhemispheric account (at least as a necessary mechanism).

How the present account differs. The SC-modulation account proposed here makes distinct predictions: (1) effects should be direction-specific (comfortable vs. uncomfortable direction), not merely task-dependent; (2) effects should be observable in SC-related physiological measures (startle, pupillary responses) specifically, not just working memory indices; (3) slow movements should be at least as effective as rapid movements, contrary to working memory taxation predictions.

A further distinction concerns directional specificity and cognitive load. If working memory taxation were the sole mechanism, any direction of eye movement imposing equivalent cognitive load should produce similar effects. However, BCT’s emphasis on client-selected ‘comfortable directions’ which vary idiographically across individuals and sessions suggests sensitivity to the qualitative nature of sensory feedback rather than quantitative cognitive demand. This directional preference cannot be straightforwardly derived from working memory accounts. Additionally, BCT employs slow, gentle eye movements that impose minimal working memory demands compared to the rapid bilateral movements typically used in EMDR research. If working memory taxation were the primary mechanism, such low-load movements should produce correspondingly weaker effects. Yet clinical observations suggest therapeutic effects occur nonetheless. This low-load, high-effect pattern is difficult to reconcile with pure working memory accounts and points toward alternative mechanisms such as SC-mediated orienting modulation, interoceptive prediction updating, or the homeostatic action selection processes emphasized in active inference frameworks.

We acknowledge that these mechanisms are not mutually exclusive—eye movement interventions may engage multiple processes simultaneously. The value of the present framework lies not in claiming SC-modulation as the sole mechanism, but in generating specific, distinguishable predictions that can advance understanding of which components contribute to therapeutic effects under which conditions. Comparative studies directly testing these alternative accounts against the SC-modulation hypothesis are essential for theoretical progress.

### 4.4 Common factors versus technique-specific components

An important distinction must be drawn between common therapeutic factors and technique-specific components. Common factors—including therapeutic alliance, safety, expectancy, and attentional engagement—likely contribute to all body-oriented interventions (Norcross and Wampold, 2011). The present framework does not claim that BCT-specific procedures (unilateral eye movement toward comfortable direction, acupoint stimulation) are necessary for therapeutic change; rather, these procedures are hypothesized to engage the proposed mechanisms, which may also be engaged through other means. Whether technique-specific components add value beyond common factors remains an empirical question requiring dismantling studies and component analyses. The framework’s utility lies not in advocating for any particular technique but in providing testable hypotheses about mechanism engagement that can guide such comparative research.

If technique-specific components do contribute unique variance, this should manifest in specific, measurable ways. For instance, if comfortable-direction eye movements specifically modulate SC-related threat processing beyond general attentional engagement, then comparing preferred-direction versus non-preferred-direction conditions should yield differential physiological outcomes (see Prediction 4 below). Similarly, factorial designs comparing eye movement alone, acupoint stimulation alone, and their combination could isolate component contributions. Such dismantling research would advance both theoretical understanding and clinical optimization.

### 4.5 Future research directions: testable predictions

This framework generates specific, testable predictions:

Prediction 1: Interoceptive precision changes. Body-oriented interventions should alter interoceptive processing, measurable through changes in interoceptive accuracy tasks and neural correlates. We note that heartbeat detection accuracy has been critiqued as an interoceptive measure (Desmedt et al., 2018), and alternative or complementary measures should be considered, including respiratory interoception (Harrison et al., 2021), gastric interoception (Azzalini et al., 2019), or confidence-accuracy calibration approaches (Garfinkel et al., 2015).

• Operationalization: Pre-post measurement surrounding body-oriented intervention vs. active control (e.g., cognitive therapy). • Measures: Interoceptive accuracy (multiple modalities), interoceptive sensibility (MAIA-2), interoceptive confidence-accuracy correspondence, insula BOLD response using perturbation-based paradigms or computational model-based fMRI (cf. Critchley and Garfinkel, 2017). • Falsification: No group difference or superior interoceptive outcomes in control condition.

Prediction 2: SC pathway modulation. Eye movement-based interventions should modulate SC-mediated threat processing, indexed by changes in startle reflex magnitude, pupillary responses, and—where technically feasible—brainstem-sensitive fMRI measures or SC-informed task contrasts during threat processing (cf. Lanius et al., 2017; Harricharan et al., 2016; Kragel et al., 2021).

• Operationalization: Eye movement intervention vs. fixation control during trauma recall. • Measures: Acoustic startle EMG, pupillometry, brainstem fMRI (where feasible). • Falsification: No modulation of defensive physiology, or equivalent effects in fixation control.

Prediction 3: Prediction error and outcome. Clients reporting greater “positive surprise” during sessions (e.g., “I expected to feel worse than I do”) should show greater symptom reduction, testable through session-by-session process measures correlated with outcome (cf. Lane et al., 2015).

• Operationalization: Session-by-session ratings of expectancy violation (“How much better/worse did you feel than expected?”). • Measures: Expectancy violation scale, weekly symptom measures (e.g., PCL-5, IES-R). • Falsification: No correlation between positive surprise and symptom reduction, or negative correlation.

Prediction 4: Directional preference and trauma severity. If comfortable-direction preference reflects safety-related processing specifically, preference strength (the magnitude of subjective difference between directions) should correlate with trauma severity or current symptom levels.

• Operationalization: Cross-sectional assessment of direction preference strength and symptom measures. • Measures: Rating of directional preference magnitude (0–10 scale), PCL-5, dissociation measures (DES-II). • Falsification: No correlation between preference strength and symptom severity.

Prediction 5: Eye movement direction effects. If comfortable-direction eye movements specifically engage safety-related processing (rather than reflecting oculomotor ease, attentional bias, or vestibular factors), unilateral movements toward the preferred direction should produce greater physiological calming (heart rate variability increase, skin conductance decrease) than movements toward the non-preferred direction.

• Standardized exploration protocol: Participants first recall distressing material and identify an associated target body sensation (e.g., tension, discomfort, activation). With eyes closed, participants slowly move their eyes horizontally left and right several times, noticing which direction feels more comfortable. Amplitude approximately 30° from central fixation—a range corresponding to the oculomotor comfort zone and SC motor map representations. For the intervention phase, participants typically open their eyes and perform unilateral movement only toward the identified comfortable direction, synchronized with relaxed breathing rhythm (approximately one gaze excursion per breath cycle): center → comfortable direction → center, repeated for 7 cycles. Direction preference assessed via forced-choice and magnitude rating (0–10). Protocol should control for prior fatigue and head position (chin rest). These protocol parameters—3 complete left–right cycles for the initial exploration phase and 7 unilateral cycles for the intervention phase, approximately 2 s per direction, 30° amplitude—are derived from typical clinical practice in BCT rather than theoretical optimization; future parametric studies should systematically vary these parameters to identify optimal values and test whether effects are parameter-sensitive. • Operationalization: Within-subject comparison of preferred vs. non-preferred direction (counterbalanced order), with 60-s sustained gaze toward each direction. • Measures: HRV (RMSSD), skin conductance level (SCL), subjective distress (SUD 0–10), state dissociation scale; manipulation checks: ocular strain rating (0–10), neck tension rating (0–10), saccade latency, smooth pursuit gain. • Falsification: No physiological difference between directions, or opposite pattern (non-preferred more calming), would suggest comfort preference reflects factors other than safety-related processing. If physiological differences correlate primarily with strain ratings or oculomotor quality rather than direction, oculomotor factors would be implicated. • State-dependence test: Repeat assessment after trauma processing; if direction preference shifts toward neutral concurrent with symptom reduction, this supports the safety-processing hypothesis.

Prediction 6: Differential mechanism engagement. Different body-oriented techniques should show distinguishable patterns of mechanism engagement—for example, EMDR emphasizing bilateral stimulation effects (and potentially working memory taxation), Somatic Experiencing emphasizing interoceptive tracking, BCT combining eye movement with direction-specific effects—testable through comparative process research.

• Operationalization: Comparative trial with standardized process measures across different body-oriented approaches. Importantly, factorial designs should systematically isolate and combine components (eye movement alone, tactile stimulation alone, interoceptive attention alone, and their combinations) to determine whether effects are additive, synergistic, or potentially interfering—a critical methodological consideration given that multimodal stimulation does not guarantee synergistic outcomes. • Measures: Interoceptive accuracy change, defensive physiology, working memory load indices. • Falsification: Indistinguishable mechanism profiles across techniques, or profiles opposite to theoretical predictions. Prediction 7: Tactile stimulation location specificity. If acupoint-specific stimulation contributes effects beyond general rhythmic tactile stimulation, then acupoint tapping should produce greater autonomic calming than matched-intensity tapping to non-acupoint locations.

• Operationalization: Within-subject comparison of acupoint (e.g., Hegu/LI4) versus non-acupoint (e.g., dorsal hand, matched distance from wrist) tapping during trauma recall, counterbalanced order. • Measures: HRV (RMSSD), SCL, SUD; credibility/expectancy ratings as control for placebo effects. • Falsification: No difference between locations, or non-acupoint superior, would suggest acupoint specificity is not relevant and general rhythmic tactile stimulation is sufficient.

These predictions transform the framework from speculative proposal to research program, inviting empirical evaluation and refinement.

## 5 Limitations

Several limitations should be acknowledged.

First, while BCT is a relatively new approach and published peer-reviewed journal articles are currently limited, the theoretical framework presented in this paper is grounded in clinical experience. This foundation is supported by 9 years of training provided to over 900 clinicians, as well as case presentations at domestic academic conferences and public symposia in Japan. Furthermore, the approach has been adopted by a number of medical institutions and counseling centers in Japan. Nevertheless, to our knowledge, no published randomized controlled trials of BCT exist as of this writing. This paper does not claim BCT superiority over established methods, nor that the theoretical framework has been empirically verified. BCT serves as an illustrative case for articulating theoretical principles potentially contributing to broader understanding of body-oriented interventions, not as an example of an empirically validated treatment.

Second, the proposed framework is by nature a conceptual integration rather than direct empirical demonstration. The predictive processing account of body-oriented intervention remains largely theoretical, and specific mechanisms through which somatic engagement facilitates maladaptive prediction updating require further empirical investigation. Neuroimaging, psychophysiological measurement, and controlled clinical trials will be essential for testing framework-generated hypotheses.

Third, the author developed BCT, potentially introducing presentation bias. To mitigate this, the paper maintains distinction between general theoretical framework and specific clinical application, acknowledging BCT’s preliminary evidence base. Readers should note that the clinical observations reported (Section 3.3) come from an unblinded developer, limiting their evidentiary value.

Fourth, generalizability from a single, culturally specific intervention warrants caution. BCT was developed and primarily practiced in Japan, and the concept of karada ni yudaneru (“entrusting to the body”) may reflect culturally specific assumptions about the body–mind relationship. Japanese cultural concepts of embodiment—including notions such as ki (vital energy) and karada (body as lived experience rather than mere physical object)—may shape both client expectations and therapeutic process in ways that differ from Western mind–body frameworks (cf. de Ozawa-Silva, 2002). Whether the principles articulated here translate across cultural contexts, or whether the underlying mechanisms operate similarly in different cultural frameworks, has not been examined. The extent to which articulated theoretical principles apply to other body-oriented approaches remains open for future comparative research.

Fifth, the “comfortable direction” construct central to BCT has not been psychometrically validated. Basic reliability data (test–retest stability within and across sessions, inter-rater agreement when applicable) and validity evidence (correlations with established measures of threat processing, interoceptive sensitivity, or hemispheric lateralization) are currently unavailable. This represents a critical gap requiring attention before the construct can be confidently employed in empirical research. Future studies should establish whether comfortable-direction preference demonstrates adequate reliability and whether it relates meaningfully to other theoretically relevant individual differences.

Finally, regarding individual differences: the framework does not currently specify who would be most likely to benefit from body-oriented interventions. Individual differences in PTSD vulnerability and resilience may be understood within the present framework in terms of variability in precision-weighting capacity across subcortical and cortical circuits. Neurobiological factors—including amygdala reactivity, hippocampal volume, prefrontal top-down regulatory capacity, and dysregulation of the hypothalamic–pituitary–adrenal (HPA) axis—are known to moderate PTSD risk following trauma exposure. Chronic HPA dysregulation may compromise the flexibility of predictive updating by altering the neuromodulatory context within which SC-PAG circuits operate. Developmental factors, including early adversity and insecure attachment, may further shape SC-PAG circuit sensitivity during critical developmental periods. Factors such as baseline interoceptive accuracy, trauma type, or presentation (hyperarousal vs. dissociative) may moderate treatment response. These questions represent important directions for future research.

## 6 Conclusion

This paper proposed a theoretical framework for understanding body-oriented trauma intervention by integrating predictive processing theory with SC-mediated subcortical pathways. Four mechanisms—interoceptive attention, somatic safety experience, SC pathway modulation, and top-down control relaxation—are proposed as intermediate principles potentially underlying body-oriented approach effectiveness. Body Connect Therapy illustrated how specific clinical techniques may map onto these mechanisms. The framework serves as a conceptual tool for organizing clinical observations, generating testable hypotheses, and facilitating dialogue between body-oriented clinicians and the broader scientific community. While empirical validation remains necessary, this theoretical contribution may stimulate further research into body-oriented trauma intervention mechanisms and ultimately contribute to developing more effective treatments for trauma-related suffering.

## Data Availability

The original contributions presented in the study are included in the article/supplementary material, further inquiries can be directed to the corresponding author.
